# Transcriptomic Profile and Sexual Reproduction-Relevant Genes of *Alexandrium minutum* in Response to Nutritional Deficiency

**DOI:** 10.3389/fmicb.2019.02629

**Published:** 2019-11-19

**Authors:** Fan-Qiang Meng, Jun-Ting Song, Jin Zhou, Zhong-Hua Cai

**Affiliations:** ^1^School of Life Sciences, Tsinghua University, Beijing, China; ^2^Shenzhen Public Platform of Screening and Application of Marine Microbial Resources, The Shenzhen International Graduate School, Tsinghua University, Shenzhen, China

**Keywords:** marine dinoflagellates, transcriptome, *Alexandrium minutum*, nutrient limitation, mixotrophic, sexual reproduction, response mechanisms

## Abstract

*Alexandrium minutum* is a typical marine toxic dinoflagellate responsible for producing paralytic shellfish poisoning (PSP) toxins. Until now, we know little about the genomic information of *A. minutum*, so a transcriptome study was conducted to clarify the physiological adaptations related to nutritional deficiency. Here, we performed RNA-Seq analysis to assess the gene expression patterns of *A. minutum* under N and P deficient conditions for 0 (control), 6, and 72 h. Main differences between the control and experimental groups were observed in hydrolase activity and fatty acid, lipid, protein, and P metabolism. Activities of photosystem I (PSI) and PSII were significantly down-regulated, and the endocytosis pathway (clathrin-dependent endocytosis) was significantly enriched under N and P stress compared with the control, indicating that *A. minutum* shifts its trophy pattern under N and P stress. We also identified several unigenes related to the process of sexual reproduction, including sex determination, sperm-egg recognition, sex differentiation, mating, and fertilization. Approximately 50% of the successfully annotated unigenes were differentially expressed between the short-term stimulated sample (6 h) and control (R). However, the expression level of most unigenes returned to normal levels after 72 h, indicating that N and P stress plays a limited role in the induction of sexual reproduction. Furthermore, the quantitative real-time PCR (qRT-PCR) results of the five representative sex-related unigenes were consistent with sequencing data, which confirmed the authenticity of transcriptomic analysis. Also, qRT-PCR analysis showed that the long and short form transcripts of the saxitoxin biosynthesis gene (*sxtA*) were down-regulated under the nutrient deficient condition compared with the control, indicating that N and P stress regulates *sxtA* expression. Overall, transcriptome analysis of *A. minutum* revealed that N and P deficiency induced responses associated with stress response, photosynthetic efficiency, toxin biosynthesis, and sexual reproduction. Our data indicate that algae change their trophic modes (to facultative mixotrophy) and related physiological reactions under stress conditions; this possibly represents an ecological adaption strategy in the algal life cycle.

## Introduction

Harmful algal bloom (HAB) is an ecological phenomenon caused by the explosive growth and accumulation of phytoplanktons, protozoa, or bacteria in seawater (Hallegraeff, 1993; Granéli and Turner, 2006b; Zhang et al., 2018a). The formation of bloom is specific to HAB species (Paerl, 1988; Granéli and Turner, 2006a). Dinoflagellates, a representative HAB species, are haploid eukaryotic microalgae with diverse modes of growth and can be found in most aquatic habitats worldwide. Some dinoflagellates, such as *Alexandrium minutum*, produce toxins [for example, paralytic shellfish poisoning (PSP) toxins] that threaten the health of human beings. *A. minutum* can survive in a relatively wide range of temperature and salt conditions (Vila et al., 2005; Bravo et al., 2008); thus, the blooms caused by *A. minutum* are observed on a worldwide scale (Chang et al., 1997; Hwang and Lu, 2000).

The life cycle of *A. minutum* is highly complex and involves vegetative cell growth, temporary cyst formation, gamete fusion, planozygote generation, and resting cyst germination (Xiao et al., 2003; Granéli and Turner, 2006a). The temporary cyst and resting cyst are inherently different structures; the former lacks flagella and is often observed under laboratory culture conditions, while the latter is formed as a result of conjugation of two gametes and is often found in sediments. Cyst formation plays an important role in the ecology of dinoflagellates because resting cysts are able to survive harsh environmental conditions better than vegetative cells (Matsuoka and Fukuyo, 2000). The deposition of resting cysts in sediments potentially contributes to the formation and maintenance of *A. minutum* blooms. Based on their characteristics, resting cysts are considered to form the seed bed of HAB (Dale, 1983). Moreover, the formation of resting cysts is usually considered as an indicator of sexual reproduction (Figueroa et al., 2007). In the past decade, many studies have focused on cysts, including induction conditions, formation process, and ecological profiles (Anderson et al., 1987; Hardeland, 1994; Perez et al., 1998; Matsuoka and Fukuyo, 2000; Sgrosso et al., 2001; Kremp and Parrow, 2010; Zhang et al., 2018b). Among the induction conditions, nutrition is regarded as the most effective inducer of encystment (Stosch, 1973; Blanco, 1995; Binder and Anderson, 2010; Blackburn et al., 2010; Figueroa et al., 2011). Deficiency of nutrients, mainly nitrogen (N) and phosphorus (P), is reportedly a major factor that induces the formation of cysts (Blanco, 1995). The concentration of NH_4_ has been shown to promote cyst formation in *Scrippsiella trochoidea* (Wang et al., 2014).

*A. minutum* shows physiological adaptation under various environmental stresses, such as nutrient starvation. Under harsh environments, *A. minutum* changes its photosynthetic mode (autotrophic or mixotrophic), toxin release behavior, and reproduction mode (sexual or asexual) (Jeong et al., 2005a, 2010). However, direct evidence supporting the mechanism of response to nutrient deficient conditions and related biochemical reactions, is lacking.

In this study, we conducted *de novo* transcriptome sequencing of *A. minutum* under N and P deficient conditions. The aim of this study was to: (1) investigate the expression patterns of genes involved in photosynthesis and endocytosis pathways, (2) identify and analyze differences in expression levels of genes related to sexual reproduction between control and nutrient deficient conditions, and (3) understand the other molecular mechanisms (such as cell wall biogenesis and saxitoxin biosynthesis) underlying the response of *A. minutum* to nutrient deficiency, and collect evidence to prove that *A. minutum* is not a purely photosynthetic organism.

## Materials and Methods

### Sample Preparation and Collection

Non-axenic cultures of *A. minutum* were obtained from the Collection Center of Marine Bacteria and Algae, Xiamen University, China. Cultures were maintained in F/2 medium (without silicate) (Guillard, 1975). Natural seawater (33 ppt salinity) was used to prepare the F/2 medium; the seawater was obtained from Dongchong (Shen Zhen), filtered through 0.22-μm filter, and autoclaved. Cells of *A. minutum* were grown in F/2 medium at 23 ± 1°C under 12 h light/12 h dark photoperiod and 60–70 μmol photons m^−2^ s^−1^ light intensity.

*Alexandrium minutum* cells in the logarithmic phase were collected as the inoculum and transferred to 120 ml sterile dialysis bags. All dialysis bags were placed in 10 L beakers filled with artificial seawater (the method of Schleicher and Schal) without N and P. The artificial seawater was exchanged at 2-h intervals for the first 12 h. The *A. minutum* cells were harvested using a 30-μm mesh at 0, 6, and 72 h, frozen in liquid nitrogen, and stored at −80°C until needed for cDNA library construction. Samples harvested at 0, 6, and 72 h are hereafter referred to as R, S6, and S72, respectively. This experiment was performed in triplicate at each time point.

### RNA Extraction, Library Preparation, and Sequencing

Total RNA was extracted from nine samples (three treatments × three replicates) using the E.Z.N.A.® Plant RNA Kit (Omega, USA), according to the manufacturer's instructions, and treated with DNase I (Omega, USA) to remove traces of contaminating genomic DNA. RNA purity and concentration were determined using the NanoPhotometer® spectrophotometer (IMPLEN, CA, USA) and Qubit® RNA Assay Kit in Qubit® 2.0 Flurometer (Life Technologies, CA, USA). RNA quality and integrity was assessed by gel electrophoresis on 1% agarose gels and by Agilent Bioanalyzer 2100 system (Agilent Technologies, CA, USA). Only high quality RNA samples were used in subsequent experiments. Each of 1.5 μg RNA per sample was used for the RNA sample preparations. Meanwhile RNA-Seq libraries were constructed using the Illumina NEBNext® Ultra™ RNA Library Prep Kit (NEB, USA). Briefly, mRNA was purified and enriched from total RNA using poly-T oligo-attached magnetic beads. Under elevated temperature, fragmentation was carried out using NEBNext First Strand Synthesis Reaction Buffer (5X). First-strand cDNA was synthesized from the mRNA using random hexamer primer and M-MuLV Reverse Transcriptase (RNase H^−^). Subsequently, second-strand cDNA was synthesized using the first-stand cDNA template, DNA Polymerase I, dNTPs, and RNase H. To each sample, index codes were added to attribute sequences with size-selected and adaptor-ligated cDNA at 37°C for 15 min followed by 95°C for 5 min. Subsequently, PCR amplification was performed using universal PCR primers, index (X) primer, and Phusion High-Fidelity DNA polymerase. Finally, both the purified PCR products and library quality were assessed on the Agilent Bioanalyzer 2100 system. The RNA-Seq libraries were sequenced on Illumina PE150 (Hiseq X Ten) platform at the BGI-Write Genomic Company. RNA-Seq data were deposited in the National Center for Biotechnology Information (NCBI) Sequence Read Archive (SRA) database under the accession number SRP154845.

### Data Filtering and *de novo* Assembly

Prior to transcriptome assembly, reads were filtered according to strict criteria. Low quality reads and reads containing poly-N stretches and adapter sequences were removed from raw reads. After filtering, 39–62 million clean reads were obtained per sample (Table S1), which were used for downstream analyses. Additional parameters of clean reads, including GC content, Q20 and Q30 values, and sequence duplication level, were also calculated.

To analyze RNA-Seq data without a reference genome, left read1 files from the nine libraries were pooled into one left.fq file. Similarly, right read2 files from the nine libraries were pooled into the right.fq file. The left.fq and right.fq files were used to *de novo* assemble the reference transcriptome of *A. minutum* using Trinity (Grabherr et al., 2011) with min_kmer_cov set to 2.

### Functional Annotation of Genes and Quantification of Gene Expression

Gene function annotation was based on the following databases: NCBI non-redundant protein sequences (NR), NCBI non-redundant nucleotide sequences (NT), Protein Family (Pfam), euKaryotic Ortholog Groups (KOG), Swiss-Prot (a manually annotated and reviewed protein sequence database), Kyoto Encyclopedia of Genes and Genomes (KEGG) Ortholog database (KO), and Gene Ontology (GO). Based on the NR and Pfam databases, Blast2GO v2.5 was used to determine the GO classifications and terms (Götz et al., 2008). KEGG Automatic Annotation Server (KAAS) annotation (http://www.genome.jp/tools/kaas/) was used to obtain KO codes for each unigene. The open reading frames (ORFs) of unigenes were defined according to the results of BLASTX against the NR and Swiss-Prot databases. The coding sequences (CDSs) of unigenes were translated into amino acid sequences, based on genetic codes. Potential ORFs of unigenes with no hits in NR and Swiss-Prot databases were predicted using ESTScan (version 3.0.3). Amino acid sequences were also functionally annotated using the Pfam database (Finn et al., 2008), and functional domains were identified using HMMER 3.0 (http://hmmer.janelia.org/) (Zhang and Wood, 2003).

Transcripts from the assembled reference transcriptome were regarded as reference sequences. Using RSEM (Li and Dewey, 2011), clean reads of all nine libraries were individually mapped onto the reference sequences, and read counts for each gene were calculated. Considering the effect of sequencing depth and gene length on read count, the FPKM parameter, which measures the expected number of Fragments Per Kilobase of transcript sequence per Million base pairs sequenced, was calculated from read-count numbers and used for the quantification of gene expression (Trapnell et al., 2010).

### Gene Expression Analysis and Functional Enrichment

DESeq2 (Love et al., 2014) was used to analyze the differential expression of genes between control and nutrient deficient conditions. The resulting *p*-values were adjusted (*p*-adj) to control the false discovery rate. Genes with *p*-adj <0.005 and |log_2_fold-change| > 1 were considered as significantly differentially expressed.

To better understand the function of differentially expressed genes (DEGs), GO enrichment analysis and KEGG pathway analysis were performed for DEGs identified in all comparisons, i.e., S6 vs. R, S72 vs. R, and S72 vs. S6). The GOseq R package (Young et al., 2010) based Wallenius non-central hyper-geometric distribution was implemented to test DEGs in GO term functional analysis. KEGG significant enrichment analysis, which provides information on DEGs involved in biochemical metabolic pathways or signal transduction pathways, was performed using KOBAS (Mao et al., 2005).

### Expression Analysis of Sex-Related Genes by Quantitative Real-Time PCR (qRT-PCR)

The results of GO classification revealed genes related to sexual reproduction (GO:0019953, Lev3), developmental process involved in reproduction (GO:0003006, Lev3), and single organism reproductive process (GO:0044702, Lev3). In this study, the description of GO terms (Lev4), including sex determination, sperm-egg recognition, sex differentiation, mating, and fertilization were used for differential gene expression analysis. The FPKM values of all DEGs identified in all three comparisons were used for hierarchical cluster analysis. The heatmap was created using the “pheatmap” package of R.

Five different expression sex-related unigenes (Cluster-20764.3338, Cluster-20764.90078, Cluster-19031.0, Cluster-9927.0 and Cluster-20764.31914) were performed by qRT-PCR to validate the expression of high-throughput sequencing, and primers of these unigenes have been displayed in Table 1. The β-actin gene (internal control) was amplified using β-actin-F and β-actin-R primers (Table 1). The qRT-PCR was conducted on ABI PRISM® 7300 Real Time PCR System using SYBR Premix Ex Taq II (Tli RNaseH Plus) Kit. The PCR was carried out in a total volume of 25 μl, containing 12.5 μl of 2X SYBR® Premix Ex TaqTM II, 2 μl cDNA template, 1 μl each of forward and reverse primer (10 μM each), 1 μl ROX Reference Dye I (50X), and 8.5 μl double distilled water. Cycling conditions were as follows: initial denaturation at 95°C for 30 s, followed by 30 cycles of 95°C for 5 s and 60°C for 31 s. The expression of the five unigenes were normalized relative to that of β-actin. Each sample was performed in triplicate. Dissociation curve analysis was performed after each assay to determine target specificity. The baseline was set automatically by the software, and the expression of *sxtA* was determined using the 2^−ΔΔCT^ method (Livak and Schmittgen, 2001).

**Table 1:** Primers used in this study.

**Name**	**Primers sequences (5^**′**^-3^**′**^)**
3338-F	AGTTGTTTGCTCCCCTCAG
3338-R	GAACGACACTCCCTTTAAT
90078-F	CGCTCTGCTACGTCTTCA
90078-R	CACCACCATCCAATCCTC
19031.0-F	ACGGAAAGATAATAAGAGGC
19031.0-R	TTACAGCAAGGGAAAGGTGA
9927.0-F	CGGCGACTGAGAAGGGAGT
9927.0-R	CGTGGGTCAAGAACGGGAT
31914-F	AGAACAAACAGTGTCCGCTT
31914-R	CAGTCTTGGCACCAAATTAA
SxtA-LRT-F	GACATAAACGCCCACGACT
SxtA-LRT-R	GCCTCAAACAAGCACAGAT
SxtA-SRT-F	GCTCCCTGCTCATTCACAT
SxtA-SRT-R	GGGGTCCACCTTCTTCTTC
β-actin-F	ACGCAGATCATGTTCGAGACC
β-actin-R	CCAGGGCGATGTAGCAGAG
SxtA-longORFF1	TGGCGGAACGGTGAGATGGA
SxtA-longORFR1	CGGTGGCAAGGCAAATGAAT
SxtA-longORFF2	ATGTTGGCGCACGGGTGGAC
SxtA-longORFR2	ATCAGGTGCGCGGCGGCA
SxtA-shortORFF1	ACAACGGCATCGACTTGA
SxtA-shortORFR1	GGTGCTGGTGAACACCTG
SxtA-longORFF1	TGGCGGAACGGTGAGATGGA

### Identification of STX Biosynthesis Genes

Although several *sxt* genes have been identified in *A. minutum* 454 libraries (Stüken et al., 2011), sequences of the remaining genes related to STX biosynthesis remain inconclusive. The known STX biosynthesis genes of *Cylindrospermopsis raciborskii* T3 (Kellmann et al., 2008) were used as queries to perform BLAST searches to identify homologs and additional *sxt* genes in *A. minutum* and to understand STX biosynthesis (Hackett et al., 2013). Subsequently, the BLAST search strategy was used to identify homologous *sxtA* genes in the transcriptome of *A. minutum* based on nucleotide sequences of *Alexandrium fundyense* (strain CCMP1719) *sxtA* long (GenBank accession No.JF343239.1) and short isoforms (GenBank accession No.JF343238.1) with an e-value <0.1 (BLASTN). TBLASTN was performed using the SxtA4 protein sequence of *A. minutum* (GenBank: AIN34681.1) as a query to identify homologous transcripts. Nested primers, including SxtA-longORFF1, SxtA-longORFR1, SxtA-longORFF2, SxtA-longORFR2, SxtA-shortORFF1, and SxtA-shortORFR1, were the used to amplify the full-length *SxtA* long and short isoform genes. Meanwhile, ten different treatment timepoints were selected to determine the expression profiles of *sxtA* genes by qRT-PCR. The long and short isoforms of *sxtA* were amplified using SxtA-LRT-F/SxtA-LRT-R and SxtA-SRT-F/SxtA-SRT-R primer pairs, respectively.

## Results

### Identification and Annotation of DEGs Under Nutrient Deficient Conditions

The basic information from RNA-Seq analysis of nine *A. minutum* samples using Illumina Hiseq X-Ten is summarized in Tables S1–S4. According to GO annotation, 102,495 genes were classified into three functional categories (Figure S1). KOG annotation classified 36,336 unigenes into 25 categories (Figure S2). Additionally, KEGG classification annotated 25,056 unigenes (13.1%) as involved in at least one of the 128 pathways in five major metabolic pathways (human diseases were not included) (Figure S3).

**Figure 1:**
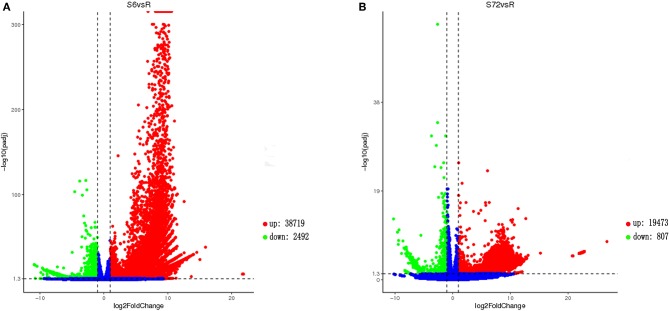
Volcano plot showing gene expression levels in *Alexandrium minutum* exposed to N and P deficient conditions for 0 (control), 6, and 72 h. **(A)** Comparison between S6 and control (R) groups. **(B)** Comparison between S72 and R groups. Down-regulated and up-regulated genes are shown in green and red, respectively (*p*-adj < 0.05; |log_2_fold-change| > 1). Genes with no difference in expression between treatment and control are indicated in blue. S6 and S72, *Alexandrium minutum* cells exposed to nutrient deficient conditions for 6 and 72 h, respectively.

Analysis of gene expression patterns between R and S6 groups using DESeq2 indicated that 41,211 transcripts were differentially expressed (Figure 1A). Of the 41,211 DEGs, 38,719 were significantly up-regulated, and 2,492 were down-regulated in the S6 group compared with R. Comparison between R and S72 groups revealed 20, 280 DEGs, of which 19,473 were significantly up-regulated, and 807 were down-regulated in the S72 group compared with R (Figure 1B). Transcripts were ranked by log_2_fold-change values; log_2_fold-change > 1 indicated up-regulated transcripts, and log_2_fold-change < 0 indicated down-regulated transcripts relative to the control.

**Figure 2:**
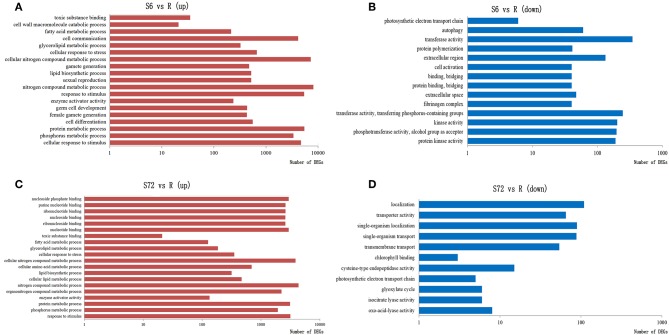
Significantly enriched GO terms in three categories (biological process, molecular function, and cellular component) in response to nutrient deficiency. GO terms of differentially expressed genes (DEGs) up-regulated in S6 **(A)**, down-regulated in S6 **(B)**, up-regulated in S72 **(C)**, and down-regulated in S72 **(D)** relative to R (control). S6 and S72, *A. minutum* cells exposed to nutrient deficient conditions for 6 and 72 h, respectively.

Transcriptome analysis of *A. minutum* revealed that nutrient deficiency for 6 h significantly up-regulated genes involved in toxic substance binding, cell wall macromolecule process, sexual reproduction, germ cell development, P metabolic process, and N compound metabolic process (Figure 2A) and significantly down-regulated genes involved in transferase activity, protein kinase activity, and photosynthetic electron transport chain (Figure 2B). Nutrient deficiency for 72 h led to significantly enriched genes involved in N compound metabolic process, single organism cellular process, chlorophyll binding, and photosynthetic electron transport chain (Figures 2C,D).

### Photosynthesis Under N and P Deficient Conditions

**Figure 3:**
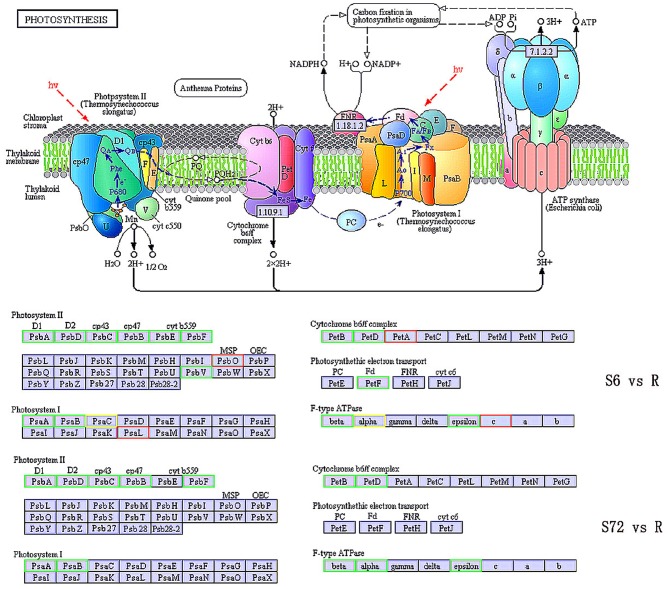
KEGG pathway enrichment analysis of photosynthesis related genes in S6, S72, and R (control) groups. Down-regulated and up-regulated genes are indicated in green and red, respectively. Both up- and down-regulated genes are shown in yellow. S6 and S72, *A. minutum* cells exposed to nutrient deficient conditions for 6 and 72 h, respectively.

Genes encoding protein subunits of photosystem I (PSI) and PSII and those encoding cytochrome (cyt) b6, cyt b6-f complex subunit 4, ferredoxin, and F-type H^+^-transporting ATPase subunits β and ε were significantly down-regulated in the S6 group compared with R. By contrast, *psaL* (encoding PSI subunit XI), *psbO* (encoding PSII oxygen-evolving enhancer protein 1), *petA* (encoding apocytochrome f), and *atpE* (encoding F-type H^+^-transporting ATPase subunit c) were up-regulated in the S6 group compared with R. Unigenes related to PsaC (PSI subunit VII) and F-type H^+^-transporting ATPase subunit α were both up- and down-regulated. In the S72 group, genes encoding proteins of PSI (PsaA and PsaB), PSII (PsbA, PsbD, PsbC, PsbB, and PsbF), cyt b6-f complex (PetB and PetD), and F-type ATPases (ATPF1B, ATPF1A, and ATPF1E) were significantly down-regulated compared with R (Figure 3).

Compared with the control group (R), the expression of *psbF*, which is involved in the regulation of KO-K02708 and encodes PSII cyt b559 subunit β, was down-regulated by ~1.1-fold in the S6 group and 7.5-fold in the S72 group. The *petD* gene, which is involved in the regulation of KO-K02637 and encodes cyt b6f complex subunit 4, was down-regulated by ~10-fold in the S6 group and 3.3-fold in the S72 group compared with R.

### Endocytosis Under N and P Depletion

**Figure 4:**
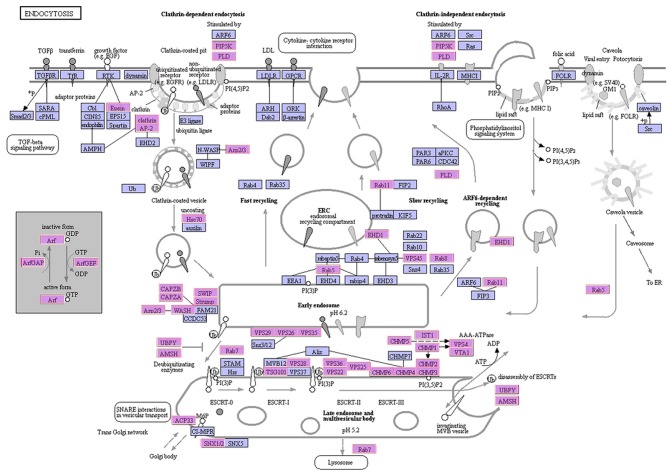
Schematic representation of the endocytosis pathway. The identified *A. minutum* proteins are indicated in pink.

Approximately 1,071 unigenes, related to 50 types of proteins, were identified in the endocytosis pathway (Table 2). Among these, unigenes related to clathrin and adaptor protein AP2 were successfully annotated. The unigenes identified in this study represented the majority of genes involved in the endocytosis pathway (Figure 4), especially those encoding clathrin, *AP-2*, and *Hsc70* (Mousavi et al., 2004). Most of the unigenes implicated in clathrin-dependent endocytosis were identified as DEGs under different experimental conditions (Figure S4). A total of 481 DEGs were involved in the endocytosis pathway, of which 470 unigenes, related to 47 types of proteins, were significantly up-regulated in the S6 group compared with R. The remaining three types of proteins (ACP33, VPS25, and CHMP6) were not encoded by DEGs. Compared with R, 5 clathrin-encoding genes, 4 AP-2-encoding genes, and 25 Hsc70 protein-encoding genes were significantly up-regulated, whereas 3 Hsc70 protein-encoding genes were significantly down-regulated in the S6 group. In the S72 vs. R comparison, 294 DEGs involved in endocytosis were identified, of which 288 unigenes were significantly up-regulated in the S72 group compared with R. Detailed information on other related functional genes is summarized in Table S5.

**Table 2:** Endocytosis related genes in *Alexandrium minutum*.

**No**	**KO ID**	**KO Description**	**Unigene number in *Alexandrium minutum***
1	K00889	1-phosphatidylinositol-4-phosphate 5-kinase	268
2	K01115	phospholipase D1/2	26
3	K03283	Heat shock 70kDa protein 1/8	138
4	K04646	Clathrin heavy chain	7
5	K05754	Actin related protein 2/3 complex, subunit 5	3
6	K05755	Actin related protein 2/3 complex, subunit 4	5
7	K05756	Actin related protein 2/3 complex, subunit 3	4
8	K05757	Actin related protein 2/3 complex, subunit 1A/1B	5
9	K05758	Actin related protein 2/3 complex, subunit 2	3
10	K07887	Ras-related protein Rab-5A	2
11	K07889	Ras-related protein Rab-5C	41
12	K07897	Ras-related protein Rab-7A	46
13	K07901	Ras-related protein Rab-8A	73
14	K07904	Ras-related protein Rab-11A	46
15	K07937	ADP-ribosylation factor 1	96
16	K10364	Capping protein (actin filament) muscle Z-line, alpha	8
17	K11824	AP-2 complex subunit alpha	4
18	K11826	AP-2 complex subunit mu-1	2
19	K11827	AP-2 complex subunit sigma-1	4
20	K11839	ubiquitin carboxyl-terminal hydrolase 8	1
21	K11866	STAM-binding protein	1
22	K12183	ESCRT-I complex subunit TSG101	4
23	K12184	ESCRT-I complex subunit VPS28	6
24	K12188	ESCRT-II complex subunit VPS22	5
25	K12189	ESCRT-II complex subunit VPS25	2
26	K12190	ESCRT-II complex subunit VPS36	2
27	K12191	Charged multivesicular body protein 2A	4
28	K12193	Charged multivesicular body protein 3	8
29	K12194	Charged multivesicular body protein 4	4
30	K12195	Charged multivesicular body protein 6	1
31	K12196	vacuolar protein-sorting-associated protein 4	16
32	K12197	Charged multivesicular body protein 1	6
33	K12198	Charged multivesicular body protein 5	9
34	K12199	Vacuolar protein sorting-associated protein VTA1	6
35	K12471	Epsin	5
36	K12479	Vacuolar protein sorting-associated protein 45	3
37	K12483	EH domain-containing protein 1	16
38	K12486	stromal membrane-associated protein	80
39	K12489	Arf-GAP with coiled-coil, ANK repeat and PH domain-containing protein	13
40	K12492	ADP-ribosylation factor GTPase-activating protein 1	22
41	K12493	ADP-ribosylation factor GTPase-activating protein 2/3	6
42	K17917	Sorting nexin-1/2	5
43	K18442	Brefeldin A-inhibited guanine nucleotide-exchange protein	20
44	K18443	Golgi-specific brefeldin A-resistance guanine nucleotide exchange factor 1	10
45	K18461	WAS protein family homolog 1	1
46	K18464	WASH complex subunit strumpellin	2
47	K18465	WASH complex subunit 7	3
48	K18466	Vacuolar protein sorting-associated protein 26	26
49	K19367	Maspardin	1
50	K19476	Vacuolar protein sorting-associated protein IST1	2

### DEGs Related to Sexual Reproduction

**Figure 5:**
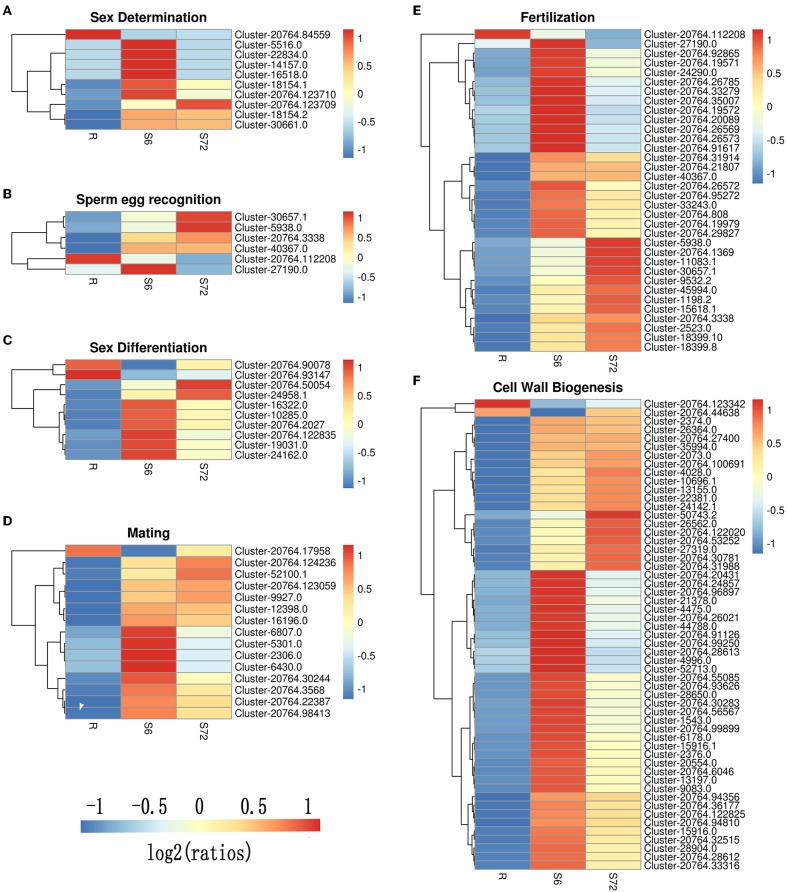
Cluster analysis of DEGs related to sexual reproduction in *A. minutum*. Six clusters were created using the “pheatmap” package of R. **(A)** Sex determination. **(B)** Sperm-egg recognition. **(C)** Sex differentiation. **(D)** Mating. **(E)** Fertilization. **(F)** Cell wall biogenesis. Specific colors represent different expression levels. Red, high expression; blue, low expression.

A total of 24 DEGs were annotated to play a role in sex determination (GO:0007530), of which 10 were significantly differentially expressed in the S6 group, and only 3 were significantly up-regulated in the S72 group compared with R (Table 3). In S6 and S72 groups, we selected two unigenes showing significant differential expression compared with the R group; one of these unigenes showed the lowest *p*-adj value, while the other unigene showed the highest log_2_fold-change value. Gene ID, *p*-adj value, log_2_fold-change value, and putative function of both unigenes are listed in Table S6. Sixteen unigenes were annotated as involved in sperm-egg recognition (GO:0035036), of which five unigenes were significantly differential expressed in the S6 group, and only two unigenes were significantly up-regulated in the S72 group compared with R. A total of 126 genes were annotated to play a role in sex differentiation (GO:0007548), of which 10 unigenes were significantly differentially expressed in the S6 group, and only 2 unigenes were significantly up-regulated in the S72 group compared with R. Of the 50 annotated genes in the mating group (GO:0007618), one unigene was significantly down-regulated and 14 unigenes were up-regulated in the S6 group compared with R, whereas only 4 unigenes were up-regulated in the S72 group compared with R. A total of 71 genes were annotated as involved in fertilization (GO:0009566), of which 33 were significantly differentially expressed in the S6 group compared with R. In the S72 vs. S6 comparison, one unigene showed significant down-regulation (Table 3). Cluster analysis was performed to examine the expression pattern of DEGs related to sexual reproduction under different experimental conditions (Figure 5).

**Table 3:** Time course distribution of up- and down-regulated genes within GO terms.

**GO term**	**The number of**	**S6vsR**	**S72vsR**	**S72vsS6**
	**annotation genes**	**up (down)**	**up (down)**	**up (down)**
sex determination	24	9 (1)	3 (0)	0 (2)
sperm-egg recognition	16	5 (0)	2 (0)	0 (0)
sex differentiation	126	8 (2)	2 (0)	0 (0)
mating	50	14 (1)	4 (0)	0 (0)
fertilization	71	33 (0)	23 (1)	0 (1)
cell wall biogenesis	218	53 (1)	31 (0)	1 (1)

**Figure 6:**
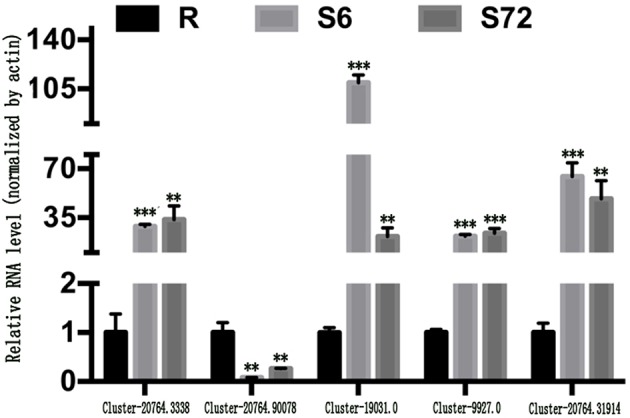
Expression of sex-related unigenes under N and P stress. Deviation bars represented the standard errors. **stand for *p* < 0.01 and ***stand for *p* < 0.001.

Five representative sex-related unigenes were selected and carried out the qRT-PCR analyses Compared with the control groups, the expression levels of all the selected unigenes under treated conditions were significantly up- or down-regulated (*P* < 0.01; Figure 6), which confirmed the authenticity of high-throughput sequencing results.

### DEGs Related to Cell Wall Biogenesis

To identify DEGs involved in cell wall biogenesis, we analyzed unigenes generated from R, S6, and S72 groups based on the comparison of |log_2_fold-change| values. Among the 218 unigenes (Table 3) mentioned above, we selected unigenes with p-adj <0.05 from R vs. S6, R vs. S72, and S6 vs. S72 comparisons. In R vs. S6 comparison, only one unigene was significantly down-regulated and 53 unigenes were up-regulated in the S6 group. In R vs. S72 comparison, 31 unigenes were up-regulated in the S72 group. In S6 vs. S72 comparison, only one unigene was up-regulated and one gene was down-regulated (Table 3). These data demonstrate that ~24.3% of the unigenes were up-regulated and 0.46% were down-regulated upon stress treatment for 6 h, and 14.2% of the unigenes were up-regulated upon stimulation with the deficiency of N and P for 72 h.

### Genes Involved in STX Biosynthesis

**Figure 7:**
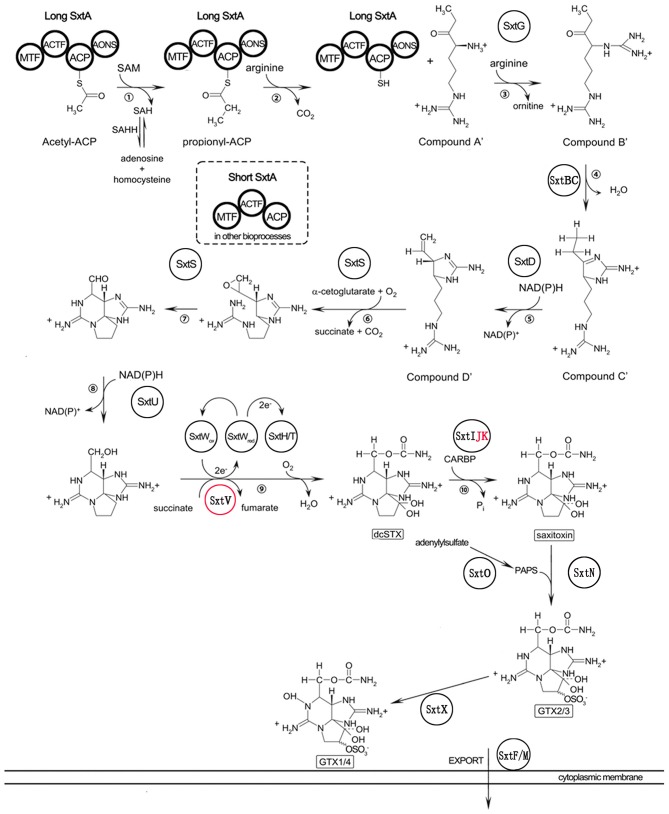
Revised saxitoxin biosynthesis pathway in *A. minutum* [cited by Kellmann et al. (2008)]. The *sxt* genes identified in this study are indicated in circles, and those not identified in this study are indicated in red letters. *Sxt* genes identified for the first time in dinoflagellates are labeled in red circles. Two types of *sxtA* genes are indicated in bold circles.

To identify genes potentially involved in STX biosynthesis, 34 annotated *Sxt* peptides from the *Cylindrospermopsis raciborskii T3* STX biosynthesis gene cluster were queried against *A. minutum* transcriptome assembled in this study using tblastn. A total of 489 homologs of 21 proteins were obtained (Table 4, and Table S7), which represented 21 of the 34 genes in *C. raciborskii*. Some of the *sxt* genes directly involved in STX biosynthesis were identified, including *sxtA, sxtD, sxtG*, and *sxtS*. Approximately 50% of the 21 genes were significantly up-regulated in the S6 group compared with R, while only 4 *sxt* genes were significantly up-regulated in the S72 group (Table S7). Furthermore, three putative homologs of *sxtV*, related to dioxygenase reductase, were first identified in dinoflagellates (Kellmann et al., 2008, 2010; Hackett et al., 2013). Based on the proposed pathway of STX biosynthesis, as previously reported, the putative STX biosynthesis gene homologs were presented in a revised STX biosynthesis pathway (Figure 7) (Stüken et al., 2011).

**Table 4:** BLAST analysis of potential STX genes in *Alexandrium minutum*.

**Gene name**	**Representive unigeneName**	**BLAST similarity match**	**GenBank**	**E-Value**	***A. minutum* Unigenes**
*orf1*	Cluster-5920.0	alr5035-like protein	ABI75088	2.00E-10	6
*sxtD*	Cluster-15500.0	Sterole desaturase	ABI75089	9.00E-14	4
*sxtB*	Cluster-20764.28596	Cytidine deaminase	ABI75093	8.00E-21	1
*sxtA*	Cluster-20764.47124	Polyketide synthase-related protein	ABI75094	3.00E-102	27
*sxtF*	Cluster-26386.0	MATE family efflux transporter	ABI75096	5.00E-08	6
*sxtG*	Cluster-25411.1	Amidinotransferase	ABI75097	5.00E-95	5
*sxtH*	Cluster-20764.93403	Phenylpropionate dioxygenase	ABI75098	6.00E-23	30
*sxtI*	Cluster-20764.31602	NodU/CmcH-related carbamoyltransferase	ABI75099	1.00E-30	3
*sxtM*	Cluster-39950.0	Sodium-driven multidrug and toxic compound extrusion protein	ABI75103	4.00E-04	5
*sxtN*	Cluster-20764.122570	Sulfotransferase	ABI75104	2.00E-08	1
*sxtX*	Cluster-20764.16179	Cephalosporin hydroxylase	ABI75105	4.00E-09	5
*sxtW*	Cluster-47915.1	ferredoxin	ABI75106	1.00E-05	18
*sxtV*	Cluster-20764.56586	FAD-dependent succinate dehydrogenase/fumarate reductase	ABI75107	2.00E-04	3
*sxtU*	Cluster-20764.94738	Short-chain alcohol dehydrogenase	ABI75108	7.00E-36	211
*sxtT*	Cluster-20764.27464	Phenylpropionate dioxygenase	ABI75109	5.00E-21	32
*sxtS*	Cluster-20764.48933	Phytanoyl-CoA dioxygenase	ABI75110	2.00E-06	6
*sxtO*	Cluster-23416.0	Adenylylsulfate kinase	ABI75115	3.00E-65	15
*sxtZ*	Cluster-20764.40105	Histidine kinase	ABI75118	1.00E-13	84
*OMPR*	Cluster-47150.0	Transcriptional regulator OmpR family	ABI75119	1.00E-35	24
*hisA*	Cluster-16520.0	HisA-related protein	ABI75120	2.00E-05	2
*orf34*	Cluster-20764.52936	Unknown	ABI75121	9.00E-20	1

*Numbers represented homologs of the Saxitoxin biosynthesis gene cluster in Cylindrospermopsis raciborskii T3*.

**Figure 8:**
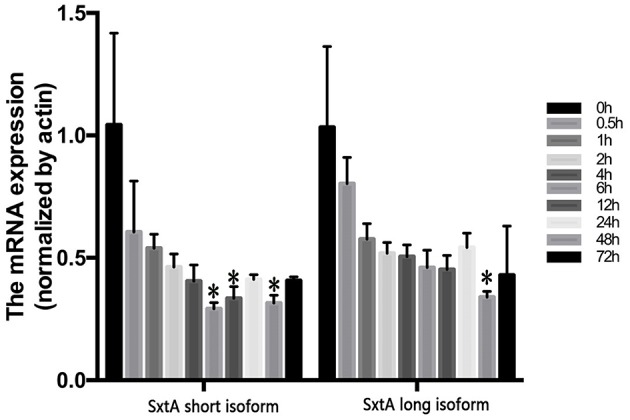
Expression of *sxtA* short and long isoform genes under N and P stress at ten time points (*p < 0.05).

Next, we used two types of *sxtA* genes of *A. fundyense* (encoding the long and short isoforms) to identify *sxtA* gene homologs in *A. minutum*. Unigenes identified using blastn were subsequently searched in the NT and NR databases for gene annotation. Nine transcripts were annotated as *sxtA* long isoform, and one transcript was annotated as *sxtA* short isoform (Table S8). Additionally, three homologous transcripts were annotated as *sxtA4* (Table S8). To examine the temporal expression profile of *sxtA* genes under nutrient deficient conditions, we performed qRT-PCR (Figure 8). The expression of *sxtA* short isoform and *sxtA* long isoform genes was down-regulated at 30 min after exposure to nutrient deficient conditions and decreased further over the next few hours. The expression of *sxtA* genes was the lowest at 6 h post treatment and then continued to increase until 24 h. Subsequently, the expression of *sxtA* genes declined until 48 h and then was slightly up-regulated at 72 h.

## Discussion

This study aimed to understand the effect of nutrient deficiency on the process of cyst formation in *A. minutum* and the expression of genes related to secondary metabolite biosynthesis. Although some studies have focused on the physical properties of dinoflagellate chromosomes (Mcewan et al., 2008), EST surveys (Bachvaroff et al., 2004; Hackett et al., 2004; Patron et al., 2005, 2006; Nosenko and Bhattacharya, 2007) and pseudogenes (Bachvaroff et al., 2008), a carefully composed transcriptome library may be more useful for the investigation of actively transcribed genes than a genome of closely related species.

### Down-Regulation of Photosynthesis in *A. minutum*

N is a major limiting factor affecting cellular biosynthesis and growth in dinoflagellates. Change in N concentration can greatly affect primary and secondary metabolism in dinoflagellates (Dagenais-Bellefeuille and Morse, 2013). N limitation causes a reduction in protein and chlorophyll levels (Lei and Song, 2011; Hockin et al., 2012; Pleissner and Eriksen, 2012; Zhang et al., 2019), resulting in the down-regulation of photosynthetic pathways, which may contribute to the reduced N demand of cells. P is one of the main limiting factors affecting algae growth. P is a component of a large number of molecules necessary for phytoplankton growth (such as DNA, RNA, and lipids), intracellular energy (e.g., ATP), and signaling pathways (Lomas et al., 2010).

In this study, genes involved in photosynthesis, particularly PSI and PSII, were significantly down-regulated under N and P deficient conditions. In conjunction with photosystem components, genes involved in light-harvesting complex II (LHCII; *lhcb1* and *lhcb2*) and ribulose-1,5-bisphosphate carboxylase/oxygenase (RuBisCO) were down-regulated under nutrient deficient conditions. This is consistent with the response of other algae to N deficiency. For example, the *lhc* genes showed 4-fold reduction in expression in *Emiliana huxleyi* under N-limitation compared with the control (Dyhrman and Anderson, 2003). In *Prymnesium parvum*, a toxin-producing microalga, N and P starvation down-regulates the expression of cyt genes (Beszteri et al., 2012). Transcriptome analysis of *A. catenella* (ACHK-T) and its non-toxic mutant form (ACHK-NT) shows the same results as our study, with up-regulated processes such as photosynthesis in ACHK-NT (Zhang et al., 2014b). Transcriptome analysis of *S. trochoidea* under N limiting condition revealed significantly inhibited photosynthesis (Cooper et al., 2016), which is consistent with our study. Reducing photosynthesis is perhaps a way to compensate for increases in cellular carbon: nitrogen (C:N) ratios; for example, in *S. trochoidea* and *A. fundyense*, the ratio of particulate organic C (POC) to PON increases under N limitation (Eberlein et al., 2016). Apoptosis of chloroplasts is a feature of resting cysts, which corresponds to the down-regulation of photosynthesis.

### Endocytosis in *A. minutum*

Autotrophy, mixotrophy, and heterotrophy are the main trophic modes of marine dinoflagellates. Like the photosynthetic species, heterotrophic dinoflagellates also contribute significantly to the ecosystem (Tillmann and Hesse, 1998; Yang et al., 2004). Various feeding mechanisms are observed in mixotrophic or heterotrophic dinoflagellates, such as diffusion feeding and filter feeding (Jeong et al., 2005b, 2010). In our study, KEGG enrichment pathway showed that genes involved in the endocytosis pathway were significantly up-regulated. We identified 190 unigenes related to 45 types of proteins that participate in the endocytosis pathway (Table 2). An intracellular vesicle is formed when a substance enters a cell. With prolonged exposure to nutrient deficient conditions (72 h), unigenes encoding clathrin, AP-2, and Hsc70 proteins were significantly up-regulated. The results indicate that the entry of nutrients in *A. minutum* cells mainly via clathrin-dependent endocytosis. Similar results have been reported in the transcriptome analysis of *A. catenella*; 131 unigenes related to 23 types of proteins involved in clathrin-dependent endocytosis were observed (Zhang et al., 2014a). This implies that *A. minutum* has a tendency to exhibit mixotrophy under nutrient deficient conditions. Thus, it is possible that clathrin-dependent endocytosis is critical for the maintenance of heterotrophy in *A. minutum*.

### Sexual Reproduction in *A. minutum*

In *A. minutum*, resting cysts are formed as a result of sexual reproduction involving the formation of gametes, followed by their fusion. Some dinoflagellate species exhibit facultative sex (Pfiester, 1989; Kremp, 2013; Figueroa et al., 2015). Cyst formation is a positive response to environmental stress such as genetic recombination, expansion of geographical distribution, and seedlings of the recurrent blooming (Dale, 1983). Cyst formation is one of the best ways for dinoflagellates to survive unfavorable conditions.

GO enrichment analysis of our transcriptome data revealed some GO terms related to sexual reproduction. During the first 6 h under N and P deficient conditions, the most significant change in expression was observed in genes encoding sex determination factors, which determine the sex of the gamete. Compared with the R group, Cluster-18154.1 showed the lowest *p*-adj value and the highest log_2_fold-change in the S6 group. Based on the NR database, Cluster-18154.1 was annotated as ubiquitin carboxyl-terminal hydrolase 22. Ubiquitin carboxyl-terminal hydrolase enzymes hydrolyze thiol esters formed between small thiols (e.g., glutathione) and the ubiquitin carboxyl terminus (Rose and Warms, 1983). These enzymes also hydrolyze glycine methyl ester and spermidine (Pickart and Rose, 1985). Cluster-18154.1 may play an important role in G protein-coupled receptor signaling pathway. Vegetative cells differentiate into male and female gametes through sperm-egg recognition. The same phenomenon is observed in the life cycle of *Chlamydomonas reinhardtii*, where vegetative cells differentiate into two types of gametes (mt^+^ and mt^−^) during N starvation (Sekimoto, 2017).

Mating and fertilization are important processes during sexual reproduction. Planktonic zygotes are formed by the fusion and mating of haploid gametes. Subsequently, diploid zygotes swim for several days before transforming into resting cysts. In this study, at 72 h under N and P starvation, 71 unigenes were annotated to play a role in fertilization, and only 1 unigene (Cluster-20764.19572) was significantly down-regulated in S72 compared with S6. The most likely function of is the fusion of sperm to the egg plasma membrane. The annotation from Pfam database indicated that it was an acrosome formation-associated factor (Afaf). Acrosome is a specialized apical vesicle of spermatozoa derived from the Golgi apparatus. Acrosome overlies the nucleus of mature spermatozoon and is formed in response to fertilization. Afaf antibodies reduce the rate of *in vitro* fertilization by significantly inhibiting the penetration of eggs by sperm (Hu et al., 2010). Therefore, down-regulation of Cluster-20764.19572 possibly enhanced fertilization. Thus, expression profiles of the abovementioned unigenes were significantly different between the control (R) and S6 groups, suggesting that *A. minutum* may carried out the ecophysiological adaption under N and P limiting conditions. With the treatment time last for 72 h, we found the expression quantity of most unigene had returned to normal level. These results revealed that nutritional deficiency might not be a strong stimulating factor for a long time. Our results were similar with other studies, i.e., nutrient deficiency alone is not enough to induce sexuality either in nature (Garcés et al., 2004; Kremp et al., 2009; Brosnahan et al., 2017) or in culture (Kremp et al., 2009; Figueroa et al., 2011).

Additionally, a thicker protective cell wall was observed during the sexual reproduction process. This suggests that genes involved in cell wall biogenesis are up-regulated in *A. minutum* to resist the adverse environment. However, it should be noted that under prolonged exposure to nutrient deficient conditions for 72 h, the expression level of most unigenes returned to the level observed at 0 h. These results suggest that nutrient deficiency does not act as an inducer of sexual reproduction over a long term.

### Toxin Production in *A. minutum*

Comparison between toxin-producing and non-toxic dinoflagellates shows that *sxtA4* is critical for toxin biosynthesis (Stüken et al., 2011). Additionally, phylogenetic analysis indicates that the C-terminal end of *sxtA* and *sxtG* plays important roles in STX biosynthesis (Hackett et al., 2013). In this study, we not only found the SXT genes related to toxin biosynthesis but also identified the *sxtV* gene. This is the first report of the identification of *sxtV* in the *Alexandrium* genus. The *sxtV* gene putatively encodes dioxygenase reductase, which belongs to the succinate dehydrogenase family. We also identified and characterized the long and short isoforms of *sxtA* gene. In the majority of toxin-producing dinoflagellates, *sxtA* produces two types of transcripts: the short transcript contains three domains (*sxtA1–3*), while the long transcript harbors four domains (*sxtA1–4*) (Stüken et al., 2015).

The long isoform of *sxtA* initiates toxin biosynthesis in cyanobacteria, indicating the long *sxtA* transcript in *A. minutum* may be directly involved in toxin biosynthesis (Kellmann and Neilan, 2007). Based on the FPKM values, homologous *sxtA4* transcripts were expressed in all experiment groups (R, S6, and S72). This suggests that the strain of *A. minutum* used for RNA-Seq analysis and transcriptome assembly may be a toxin-producing strain. Previously, analysis of two kinds of *Alexandrium ostenfeldii* (PSP toxin-producing and non-producing) strains showed that *sxtA4* and *sxtA1* fragments were not present in the non-PSP-producing strains, whereas the PSP-producing strains contained both *sxtA1* and *sxtA4* fragments (Suikkanen et al., 2013). Additionally, neither PSP nor *sxtA4* gene fragments were detected in the non-toxic *A. minutum* strain, VGO663 (Stüken et al., 2015). Compared with the non-PSP-producing *S. trochoidea* strain, neither *sxtA1* nor *sxtA4* gene fragments were detected in its transcriptome (Cooper et al., 2016). We speculate that the long isoform of *sxtA* performs the first step in toxin production. Both *sxtA* genes were down-regulated in S6 and S72 groups compared with the R group, suggesting that *A. minutum* produces less toxins under nutrient deficient conditions. These results are consistent with those of previous studies (Wang and Hsieh, 2002; Dyhrman and Anderson, 2003; Leong et al., 2004; Hu et al., 2006; Vanucci et al., 2012). The toxin contents of *Alexandrium* and *Ostreopsis ovata* decrease under N limiting conditions, possibly because algae divert more energy toward sexual reproduction under adverse environments, in order to propagate, than toward toxin production (Vanucci et al., 2012; Zhang et al., 2014b).

## Conclusions

Here, we describe the transcriptomic profiles of the toxin-producing algae of *A. minutum* under normal and nutrient (N and P) deficient conditions. Candidate genes potentially related to photosynthesis, sexual reproduction, cell wall biogenesis, and toxin biosynthesis were identified. Our results demonstrated that the significant down-regulated in photosynthesis and up-regulated in endocytosis were observed under N and P limiting conditions, which indicates that *A. minutum* is capable of switching the resource utilization pattern from trophic modes to facultative mixotrophy under the condition of nutritional deficiency. Furthermore, we also identified some unigenes related to sexual reproduction and analyzed their differential expression profile. Meanwhile, photosynthesis efficiency and toxin biosynthesis are also potentially linked to N and/or P starvation. This study provides useful data for understanding the mechanisms of ecological adaption and ecophysiological response in *A. minutum* under harsh environmental conditions.

## Data Availability Statement

The datasets generated for this study can be found in NCBI, SRP154845.

## Author Contributions

F-QM and J-TS performed the experiments, collected the samples, and analyzed the data. F-QM and Z-HC drafted the manuscript. F-QM prepared the figures and tables. F-QM, Z-HC, and JZ completed critical comments and revision.

### Conflict of Interest

The authors declare that the research was conducted in the absence of any commercial or financial relationships that could be construed as a potential conflict of interest.

## Supplementary Material

Figure S1 Gene Ontology classifications of assembled unigenes. Unigenes were assigned to three classifications: **(A)** biological processes, **(B)** cellular components and **(C)** molecular functions. In total, 102,495 unigenes with BLAST matches to known proteins were assigned to gene ontology.

Figure S2 Histogram presentation of euKaryotic Ortholog Groups (KOG) classification. A total of 36,336 unigenes were assigned the euKaryotic Ortholog Groups classification and classified into 25 functional categorie.

Figure S3 KEGG biochemical mappings for *A. minutum*. Unigenes involved in KEGG pathway were divided in five branches: **(A)** Cellular Processes, **(B)** Environmental Information Processing, **(C)** Genetic Information Processing, **(D)** Metabolism, **(E)** Organismal Systems.

Figure S4 The KEGG pathway enrichment of endocytosis in *A. minutum*. Differentially expressed genes (between S6 and R group, S72 and R group) in red (up-regulated) and yellow (both up and down regulated) have shown in endocytosis pathway.

Table S1 Overall statistical results of transcriptome sequencing for nine samples.

Table S2 The distribution of splicing length frequency.

Table S3 The distribution of splicing length.

Table S4 Annotation results of unigenes against different databases.

Table S5 Enrichment result of endocytosis pathway in different experimental groups.

Table S6 Information of representative significant differentially expressed unigenes of sex determination, sperm-egg recognition, sex differentiation, mating, fertilization and cell wall biogenesis.

Table S7 TBLASTN search of candidate STX genes and differential expression genes in the *A. minutum* transcriptome. Each sign of “TBLASTN 2.2.28+” represents an independent blast for 34 cyanobacterial proteins.

Table S8 BLAST result of *sxtA* long/short form gene and *sxtA4* domain sequence.
