# Supplementary figures and images for: Transcriptomic Profile and Sexual Reproduction-Relevant Genes of Alexandrium minutum in Response to Nutritional Deficiency
Source: Front Microbiol. 2019 Nov 19;10:2629. doi: 10.3389/fmicb.2019.02629 (PMC6877688; doi:10.3389/fmicb.2019.02629)

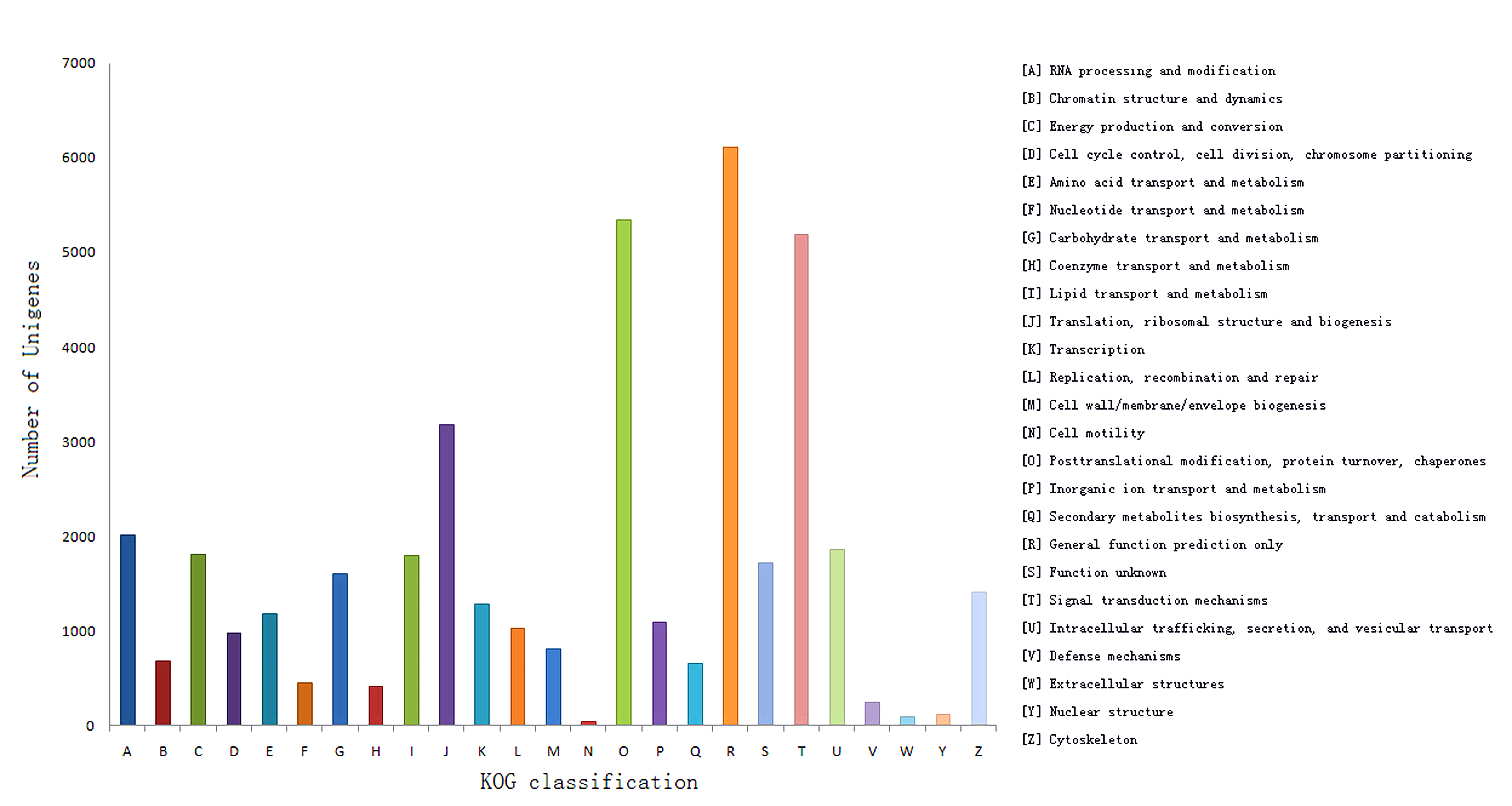

Supplement: Figure S1 — Gene Ontology classifications of assembled unigenes. Unigenes were assigned to three classifications: (A) biological processes, (B) cellular components and (C) molecular functions. In total, 102,495 unigenes with BLAST matches to known proteins were assigned to gene ontology. [file Data_Sheet_1.zip › supplementary material/supplementary figure 2.JPEG]

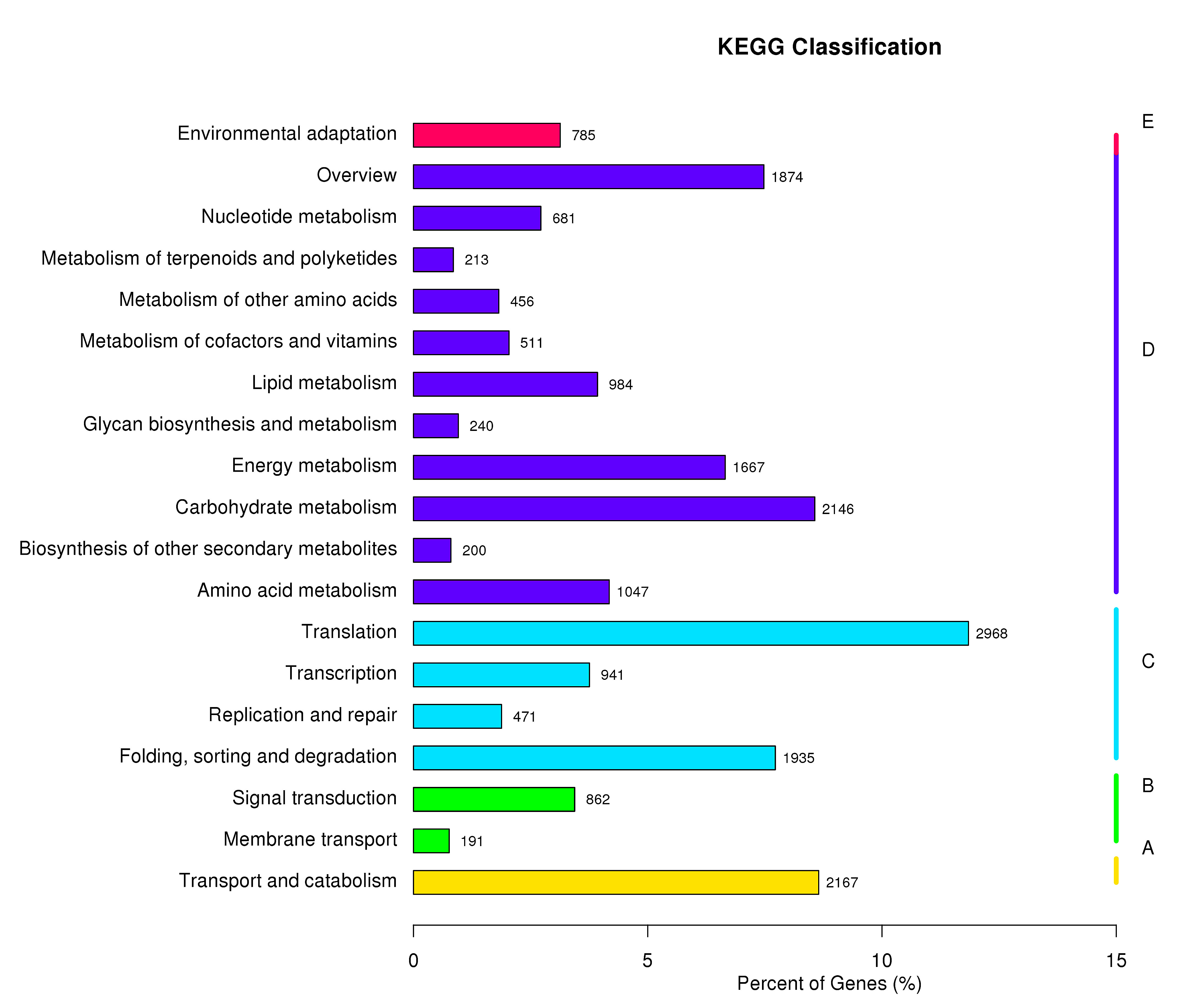

Supplement: Figure S1 — Gene Ontology classifications of assembled unigenes. Unigenes were assigned to three classifications: (A) biological processes, (B) cellular components and (C) molecular functions. In total, 102,495 unigenes with BLAST matches to known proteins were assigned to gene ontology. [file Data_Sheet_1.zip › supplementary material/supplementary figure 3.JPEG]

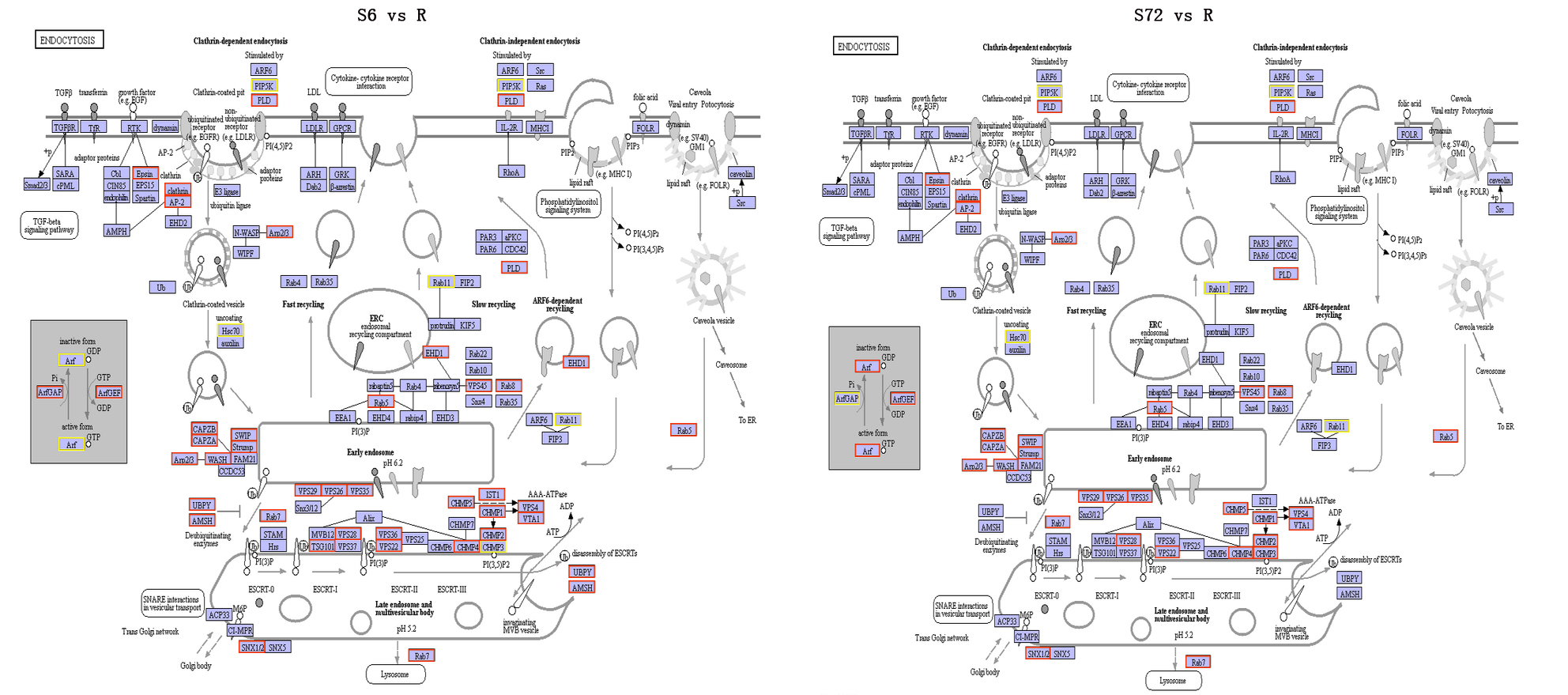

Supplement: Figure S1 — Gene Ontology classifications of assembled unigenes. Unigenes were assigned to three classifications: (A) biological processes, (B) cellular components and (C) molecular functions. In total, 102,495 unigenes with BLAST matches to known proteins were assigned to gene ontology. [file Data_Sheet_1.zip › supplementary material/supplementary figure 4.JPEG]
