# Supplementary material for: Transcriptomic Profile and Sexual Reproduction-Relevant Genes of Alexandrium minutum in Response to Nutritional Deficiency
Source: Front Microbiol. 2019 Nov 19;10:2629. doi: 10.3389/fmicb.2019.02629 (PMC6877688; doi:10.3389/fmicb.2019.02629)
Supplement: Figure S1 — Gene Ontology classifications of assembled unigenes. Unigenes were assigned to three classifications: (A) biological processes, (B) cellular components and (C) molecular functions. In total, 102,495 unigenes with BLAST matches to known proteins were assigned to gene ontology. [file Data_Sheet_1.zip › supplementary material/supplementary table 1-4.doc]

**Supplement Tables**

**Table S1**. Overall statistical results of transcriptome sequencing for nine samples

| Sample | Raw Reads | Cleanreads | Q20(%) | Q30(%) | GC(%) |
| --- | --- | --- | --- | --- | --- |
| R1 | 63519044 | 62711274 | 98.15 | 94.81 | 65.68 |
| R2 | 50271016 | 49550924 | 98.12 | 94.75 | 65.70 |
| R3 | 55966680 | 55115556 | 98.49 | 95.65 | 65.75 |
| S6_1 | 50767786 | 50010668 | 98.19 | 94.87 | 56.56 |
| S6_2 | 56789166 | 55687280 | 98.64 | 95.96 | 57.24 |
| S6_3 | 53292842 | 52647440 | 98.10 | 94.69 | 58.20 |
| S72_1 | 55172190 | 54402648 | 97.98 | 94.46 | 65.21 |
| S72_2 | 49721024 | 48858056 | 98.35 | 95.23 | 63.74 |
| S72_3 | 40462832 | 39649498 | 98.44 | 95.36 | 53.10 |

**Table S2**. The distribution of transcript length frequency

| Transcript length interval | 200-500bp | 500-1kbp | 1k-2kbp | >2kbp | Total |
| --- | --- | --- | --- | --- | --- |
| Number of transcript | 111548 | 53446 | 61672 | 32899 | 259565 |
| Number of genes | 48588 | 48431 | 61261 | 32864 | 191144 |

**Table S3**. The distribution of transcript length

|  | Min Length | Mean Length | Median  Length | Max Length | N50 | N90 | Total Nucleotides |
| --- | --- | --- | --- | --- | --- | --- | --- |
| transcripts | 201 | 1007 | 637 | 19677 | 1620 | 404 | 261365571 |
| genes | 201 | 1253 | 984 | 19677 | 1747 | 628 | 239461401 |

**Table S4**. Annotation results of unigenes against different databases

|  | Number of Genes | Percentage(%) |
| --- | --- | --- |
| Annotated in NR | 80844 | 42.29 |
| Annotated in NT | 13036 | 6.81 |
| Annotated in KO | 25056 | 13.1 |
| Annotated in Swiss Prot | 64729 | 33.86 |
| Annotated in PFAM | 102169 | 53.45 |
| Annotated in GO | 102495 | 53.62 |
| Annotated in KOG | 36336 | 19 |
| Annotated in all Databases | 6366 | 3.33 |
| Annotated in at least one Database | 121363 | 63.49 |
| Total Unigenes | 191144 | 100 |
